# *Xenopus* Oocytes as a Powerful Cellular Model to Study Foreign Fully-Processed Membrane Proteins

**DOI:** 10.3390/membranes12100986

**Published:** 2022-10-11

**Authors:** Isabel Ivorra, Armando Alberola-Die, Raúl Cobo, José Manuel González-Ros, Andrés Morales

**Affiliations:** 1 Departamento de Fisiología, Genética y Microbiología, Universidad de Alicante, Apdo 99, E-03080 Alicante, Spain; 2 Instituto de Investigación, Desarrollo e Innovación en Biotecnología Sanitaria de Elche (IDiBE), Universidad Miguel Hernández, E-03202 Elche, Spain

**Keywords:** *Xenopus* oocytes, microtransplantation method, membrane proteins, lipid-protein interactions, functional assays of mature proteins

## Abstract

The use of *Xenopus* oocytes in electrophysiological and biophysical research constitutes a long and successful story, providing major advances to the knowledge of the function and modulation of membrane proteins, mostly receptors, ion channels, and transporters. Earlier reports showed that these cells are capable of correctly expressing heterologous proteins after injecting the corresponding mRNA or cDNA. More recently, the *Xenopus* oocyte has become an outstanding host–cell model to carry out detailed studies on the function of fully-processed foreign membrane proteins after their microtransplantation to the oocyte. This review focused on the latter overall process of transplanting foreign membrane proteins to the oocyte after injecting plasma membranes or purified and reconstituted proteins. This experimental approach allows for the study of both the function of mature proteins, with their native stoichiometry and post-translational modifications, and their putative modulation by surrounding lipids, mostly when the protein is purified and reconstituted in lipid matrices of defined composition. Remarkably, this methodology enables functional microtransplantation to the oocyte of membrane receptors, ion channels, and transporters from different sources including human post-mortem tissue banks. Despite the large progress achieved over the last decades on the structure, function, and modulation of neuroreceptors and ion channels in healthy and pathological tissues, many unanswered questions remain and, most likely, *Xenopus* oocytes will continue to help provide valuable responses.

## 1. Background

**Figure 1:**
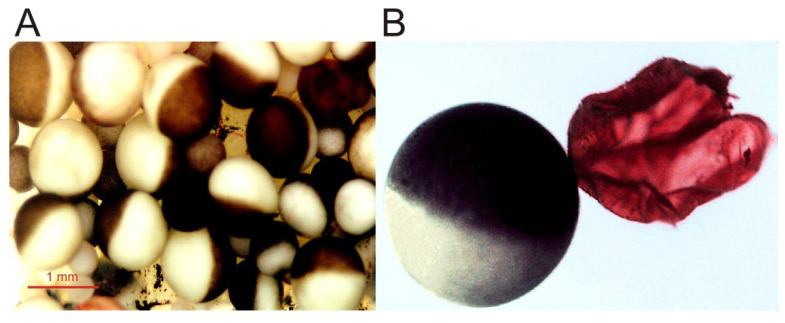
Micrographs of *Xenopus* oocytes. (**A**) Image of an opened and extended fragment of the *Xenopus* ovary lobule, showing oocytes at different stages of development. (**B**) Full-grown, immature oocyte, and the plasma membrane (together with its vitelline envelope) from another oocyte, manually isolated and stained with ink for better observation.

*Xenopus laevis*, the South African clawed frog, is a laboratory animal that is especially suitable for diverse biological studies since, unlike other anurans, their ovaries always contain oocytes in all stages of development (Figure 1) and new cycles of oogenesis can be initiated during any season by hormonal treatment. In the last five decades, the use of *Xenopus* oocytes has grown exponentially and has become a chief tool in many laboratories around the world in studies dealing with molecular, cellular, and developmental biology [1,2,3,4,5]. In fact, since the last decade of the 20th century, the *Xenopus* oocyte constitutes one of the most widely used cell model in molecular biology [3,4]. Furthermore, *Xenopus* oocytes are particularly useful cells to carry out electrophysiological recordings to study both native ion channels and receptors [6,7] (reviewed in [2,8,9,10]) and foreign proteins transplanted to this convenient host cell after the injection of either the corresponding mRNA or lipid vesicles containing the purified protein (reviewed in [2,9,11,12,13]). *Xenopus* oocytes have also become the reference model for biophysical studies dealing with structure to function relationships, addressed to unravel the functional consequences of specific mutations of membrane receptors, channels, and transporters (reviewed by [13,14]). Moreover, transplanting foreign, fully-processed membrane proteins to *Xenopus* oocytes is a methodology more recently used to look deeper into the role played by ion channels and neuroreceptors in the pathogenesis of different diseases as well as for drug screening using electrophysiological recordings and/or fluorescence assays [13,15,16]. Altogether, this has led to the *Xenopus* oocyte being regarded as a “living test tube” [17].

## 2. Advantages and Drawbacks of Using *Xenopus* Oocytes to the Study of Membrane Proteins

As stated by August Krogh [18], an outstanding physiologist awarded the Nobel Prize, for every biological problem there is an organism (model) on which it can be most conveniently studied. In this sense, the *Xenopus* oocyte model, introduced by Ricardo Miledi, constitutes an excellent paradigm of the Krogh principle to carry out detailed studies on the function of foreign membrane proteins, as was the squid giant axon to understand the ionic basis of the action potential, the frog end-plate junction and the squid stellate ganglion to unveil the basis of synaptic transmission or the *Aplysia* ganglia, which allowed for the molecular mechanisms of learning and memory to be dealt with. Some of the advantages that the oocyte fulfills to study foreign proteins are:

(a) *Xenopus* oocytes have a continuous and asynchronous development, allowing a year-round use of these cells [1].
(b) Large size, up to 1.3 mm, and roughly spherical shape [1], which facilitates electrophysiological recordings, even allowing the introduction of additional micropipettes for microinjecting compounds [2]. Furthermore, its size enables simultaneous biochemical and optical techniques with quite high spatio-temporal discrimination [19].
(c) Oocytes constitute an excellent factory for the adequate synthesis and processing of most heterologous proteins [20,21,22,23] (reviewed in [2,13,14,24,25,26,27]).
(d) These cells are easy to manage and cheap to maintain. Moreover, these cells can be kept alive in an inorganic buffer for long periods (up to several weeks) after their isolation from the ovary, without the need of a specific sterile serum or medium [2,14,16]. Nevertheless, oocytes progressively uncouple from the surrounding follicular cells after their separation from the ovary, losing responsiveness to certain hormones and neurotransmitters.
(e) The osmotic water permeability of oocytes is quite low (circa 4 × 10^−4^ cm/s), which allowed for the first functional characterization of exogenous aquaporins [28,29].
(f) The oocyte plasma membrane can be quite easily isolated either manually ([30,31]; see Figure 1) or by simple biochemical procedures [32]. The isolation of plasma membranes enables one to determine the presence of the microtransplanted protein at the oocyte plasma membrane [31,33] and allows studies concerning both the targeting of membrane proteins and the quantitation of the ratios between functional and the total number of ion channels/receptors incorporated into the cell membrane [12,33,34].
(g) Although the membrane of *Xenopus* oocytes might present certain ion channels and receptors, the expression of endogenous proteins from their own mRNA is low (about 5% of their stored mRNA, [13]). Thus the oocyte plasma membrane usually lacks significant levels of neuronal voltage-dependent Na^+^ and K^+^ channels and many neuroreceptors and transporters [10].
(h) The oocyte has powerful intracellular signaling cascades, mostly involving InsP_3_ synthesis and Ca^2+^ release from intracellular stores after phospholipase-C activation. Thus, heterologous expression of certain metabotropic receptors can be simply monitored by recording Ca^2+^-dependent Cl^−^ currents, since the Ca^2+^-dependent Cl^−^ channel, anoctamin 1 (TMEM16A), is highly expressed in the oocyte membrane [2,27,35,36].
(i) Oocytes are convenient cells to screen potential drugs and to determine their relative efficacies against specific targets. These studies have been boosted by the introduction of devices for the automated voltage-clamping of these cells [13,16].

Nevertheless, *Xenopus* oocytes also present some disadvantages when studying membrane proteins including:

(a) In the ovary, oocytes are found as follicles, constituted by several cellular and acellular layers surrounding the oocyte, from inner to outer: (i) the oocyte; (ii) a fibrous vitelline membrane; (iii) a monolayer of follicular cells, which is coupled to microvilli of the oocyte membrane by gap junctions [37]; (iv) a theca, containing mainly collagen, fibrocytes and small blood vessels; and (v) a layer of epithelial cells. The layers surrounding the oocyte might constitute a handicap to study certain transplanted proteins since follicular cells express ion channels and receptors of their own and are electrically coupled to the oocyte. However, the layers surrounding the oocyte can be removed either manually or by enzymatic means.
(b) There is a certain variability in the expression efficiency of oocytes, which has been related to seasonal differences [27]. Actually, some laboratories stop working with oocytes during summer because of their low quality. Furthermore, noise and other vibrations should be restricted around aquarium facilities since *Xenopus* are quite sensitive to them.
(c) As previously mentioned, oocytes randomly express certain ion channels and receptors, which might be confused with those heterologously expressed [13]. Thus, it becomes necessary to keep in mind the channels and receptors that can be endogenously expressed by oocytes (excellent reviews regarding this issue are provided in [2,8,9,10,24,25]), though some of them can be pharmacologically blocked. Nevertheless, other cell models commonly used for the heterologous expression of ion channels and transporters such as human embryonic kidney cells (HEK) also have endogenous ion channels [38].
(d) The large size of the oocyte together with the presence of several surrounding layers (cellular and acellular) constitute a limitation to obtaining fast ligand-applications, particularly when using large molecules [39], and consequently, to record fast-kinetics currents (it hinders the resolution of ligand-elicited currents at resolutions below hundred milliseconds; [40]). Moreover, when follicular cells remain attached to the oocyte, certain measured pharmacological values (i.e., half maximal effective concentration, EC_50_, or half maximal inhibitory concentration, IC_50_) might be inaccurate [39]. In addition, several factors such as the oocyte large size, the presence of numerous microvilli at its plasma membrane, and its electrical coupling to follicular cells contribute to eliciting extremely large capacitive artifacts, which prevent resolving fast voltage-dependent currents below a few milliseconds [14].

## 3. Transplant of Fully Processed Membrane Proteins to the *Xenopus* Oocyte

As previously indicated, *Xenopus* oocytes have allowed for the biophysical characterization of many ion channels, neurotransmitter receptors, and transporters thanks to their ease of use, amenability for electrophysiological recordings and their capability to efficiently and faithfully translate most heterologous mRNAs (reviewed in [2,24,25]). It is known that *Xenopus* oocytes are able to make many post-translational modifications of the proteins coded by the exogenous mRNA (as glycosylation, phosphorylation, acetylation, or folding), and to correctly assemble oligomeric receptor/channel complexes. However, occasionally, they do not mimic the post-translational modifications carried out by the cells that used to express them natively. Most likely, these differences account for the failed or altered function of some foreign proteins expressed in oocytes and, perhaps, also for the variability observed between oocytes [27]. For instance, *Torpedo* nicotinic acetylcholine (ACh) receptors (nAChRs) expressed in oocytes after injecting the corresponding mRNAs have an altered pattern of glycosylation [41], and neuronal sympathetic nAChRs (α3β4 and α4β4 subunits) do not exhibit certain properties of the native receptors, putatively because oocytes fail to correctly assemble their different subunits or because of the post-translational channel modifications [42]. Similarly, native brain and heterologously expressed rat α4β2 nAChRs showed significant pharmacological differences [43]. In this regard, it should be considered that certain membrane proteins including ion channels are modulated by their interaction with accessory subunits, which might be lacking in the host cell membrane and are not incorporated when mRNA is used to express specific foreign proteins in the oocyte [13,27,43,44]. Additionally, specific lipid requirements of certain membrane proteins might constitute a severe handicap to the faithful functional expression of some heterologous proteins [45]. Moreover, it has been shown that different mutations in the M4 (the outermost lipid-facing α-helix of the transmembrane domain) of α4β2 nAChRs reduced or abolished the function of these receptors when expressed in the HEK cells, but not when expressed in *Xenopus* oocytes [46]. The differences in the functional activity of these mutant nAChRs between both cell types seem to be due to the different lipid composition of their respective plasma membranes [46]. 

The above-mentioned handicaps were overcome when Marsal et al. [47] found that foreign membranes carrying neurotransmitter receptors and ion channels could be incorporated into the *Xenopus* oocyte membrane, which allowed for the microtransplantation of fully processed proteins retaining their natural environment. Since then, several groups have followed this approach of the intracellular injection of plasma membranes to study foreign membrane proteins in *Xenopus* oocytes (reviewed in [12]). Thus, many neuroreceptors, transporters, and ion channels have been transplanted from their original cells to the oocyte membrane including: (i) nAChRs from *Torpedo* electroplaques [33,47], muscle fibers [48,49], brain neurons [50], and from cell lines overexpressing heterologous neuronal nAChRs [26]; (ii) gamma-aminobutyric acid receptor type A (GABA_A_R) from the brain synaptosomal membranes [15,26,51,52,53,54,55,56,57,58,59,60,61]; (iii) glutamate (AMPA, α-amino-3-hydroxy-5-methyl-4-isoxazolepropionic acid/Kainate/NMDA, N-methyl-D-aspartate) receptors [26,52,62,63,64]; (iv) voltage-activated Na^+^ and Ca^2+^ channels from the human brain [34,65]; (v) Cl^−^ channels from *Torpedo* electroplax [33,47] and from human syncytiotrophoblast microvillous membranes [66]; and (vi) membrane transporters such as P-glycoprotein [31] and the Cl^−^ transporters, KCC1 (K^+^–Cl^−^ cotransporter type 1) and NKCC2 (Na^+^–K^+^–Cl^−^ cotransporter type 2; [55]).

**Figure 2:**
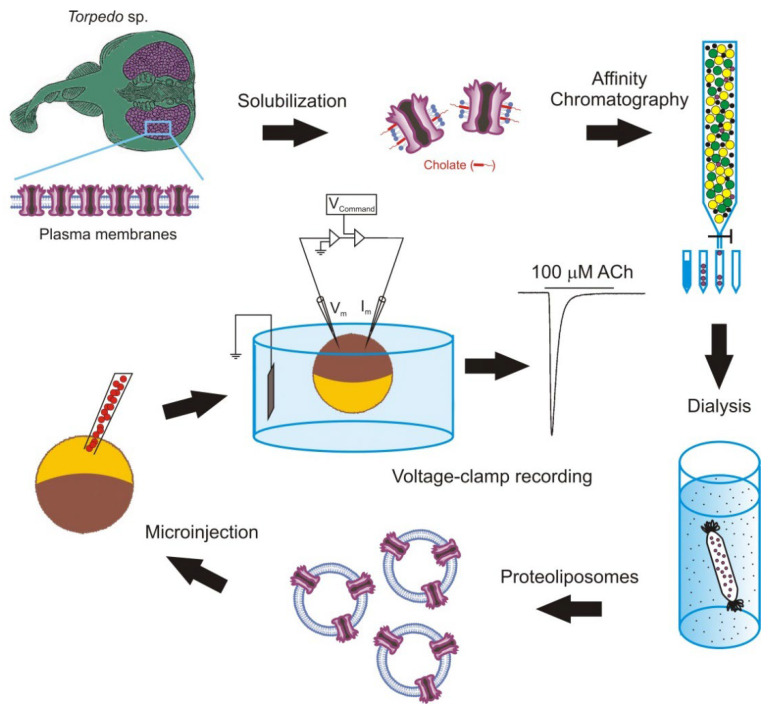
Methodology followed to microtransplant foreign membrane proteins to oocytes. Scheme of the steps to microtransplant the purified and reconstituted nicotinic acetylcholine (ACh) receptors (nAChRs) to the *Xenopus* oocyte membrane to carry out detailed functional studies.

The intracellular injection of plasma membranes to insert foreign proteins into the oocyte membrane was later extended by microtransplanting functional proteins after their purification and reconstitution in lipid vesicles of defined composition [33,67,68,69]. The main steps followed to microtransplant purified and reconstituted nAChRs from *Torpedo* electroplaques to the *Xenopus* oocyte membrane are shown in Figure 2. 

It should be noted that the goal of this experimental approach is not to use oocytes as a factory to generate proteins, but as a convenient cellular system to carry out detailed functional studies of the transplanted membrane proteins. Nevertheless, the use of purified and reconstituted proteins, instead of fragments of cellular membranes, has several advantages including: (i) it allows for the study of single molecular entities; (ii) it does not require the transplanted protein to be highly expressed in the plasma membrane, although the presence of a large amount of protein simplifies its purification; and (iii) it makes it possible to study the influence that the lipid composition of the reconstitution matrix has on both the function of the transplanted protein and the process of fusion between the vesicular and cellular membranes. This later point is of special relevance since many proteins need to interact with specific lipids for developing their full functional activity [70,71]. Consequently, the microtransplantation of purified and reconstituted proteins into the *Xenopus* oocyte membrane arises as an excellent way to unravel the lipid–protein interactions, since it allows us to both insert proteins bound to specific lipids, which can even be labelled, and to selectively modify the lipid content of the cell membrane. Using this approach, it is possible to change not only the ratio of different phospholipids surrounding the protein to determine their functional relevance, but also the charge or the length of the acyl chains to induce local changes in the bilayer thickness and elasticity, which might also be important for the protein activity [72,73]. Thus, this approach constitutes a very useful extension to the classical use of cDNA or mRNA for the functional study of ion channels and neurotransmitter receptors.

### 3.1. Characteristics of Protein Microtransplantation to the Oocyte Plasma Membrane

#### 3.1.1. Proteoliposome-Plasma Membrane Fusion Does Not Require an Intracellular Ca^2+^ Increase

**Figure 3:**
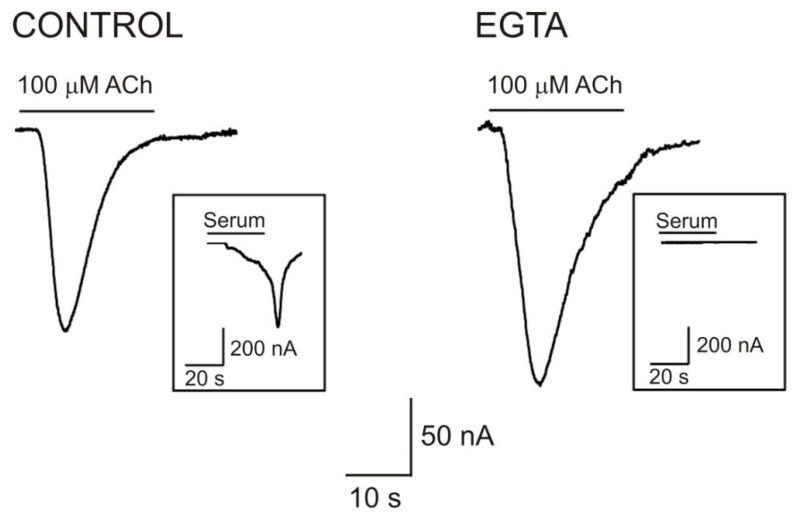
The microtransplantation of proteoliposomes to the *Xenopus* oocyte membrane does not depend on the increase in the intracellular calcium concentration ([Ca^2+^]_i_). ACh-elicited currents in oocytes microinjected with proteolipomes bearing nAChRs in the control oocytes (**left**, control) and in oocytes previously loaded with ca. 5 nM EGTA to chelate [Ca^2+^]_i_ (**right**). Note the slower desensitization on nAChRs in the EGTA loaded cell.

Several factors have been proposed to facilitate the fusion of artificial and cellular membranes including osmolarity, pH, lipid composition, and particularly, the presence of some “fusion factors” including certain proteins and Ca^2+^. Lipid bilayers can fuse together passively in the absence of Ca^2+^, but many membrane-fusion processes such as neurotransmitter and hormonal secretion, myoblast fusion, membrane resealing, and nuclear vesicle fusion in *Xenopus* eggs either require or are boosted by an increase in the intracellular Ca^2+^ concentration ([Ca^2+^]_i_). The role played by [Ca^2+^]_i_ in the efficacy of transplanting purified proteins into the *Xenopus* oocyte membrane has been explored [74]. In these experiments, [Ca^2+^]_i_ was decreased by using Ca^2+^ chelators, either through preincubating oocytes with 1,2-bis (2-aminophenoxy)ethane N,N,N′,N′-tetraacetic acid acetoxymethyl ester (BAPTA-AM) or loading the cells with ethyleneglycol-bis (β-aminoethylether)-N,N,N′,N′-tetraacetic acid (EGTA), and several hours later, these cells were injected with proteoliposomes bearing nAChRs. Effective Ca^2+^ chelation was proven by the lack of oscillatory Ca^2+^-dependent Cl^−^ currents after superfusing these cells with serum, which activates lysophosphatidic acid receptors of the oocyte membrane [75,76] and, subsequently, the phosphatidylinositol cascade (Figure 3; [74]). These experiments evidenced that both the control and Ca^2+^-chelated oocytes presented similar ACh-elicited currents (*I_ACh_*s), indicating that the fusion of the proteoliposome vesicle with the cellular membrane was not dependent on the local increases of [Ca^2+^]_i_. Nevertheless, the *I_ACh_*s elicited in oocytes loaded with either BAPTA-AM or EGTA were slightly smaller than those in the control cells. A lower incorporation of neuroreceptors after synaptosomal membrane injection has also been reported when the oocytes were loaded with Ca^2+^-chelators [61]. It has been suggested that the smaller responses might be related to a slower trafficking of lipid vesicles or, alternatively, to alteration in the binding of the injected vesicles to the plasma membrane [74]. Although a [Ca^2+^]_i_ increase must occur when rupturing the oocyte membrane by the needle when injecting the proteoliposomes, it seems that this transient [Ca^2+^]_i_ increase does not account for the fusion of proteoliposomes to the oocyte membrane. In concordance with this, the incorporation of new nAChRs to the oocyte membrane takes place for many hours after proteoliposome injection, when [Ca^2+^]_i_ should be returned to very low levels in the Ca^2+^-chelated groups.

#### 3.1.2. Microtransplanted Proteins Are Incorporated in Patches

**Figure 4:**
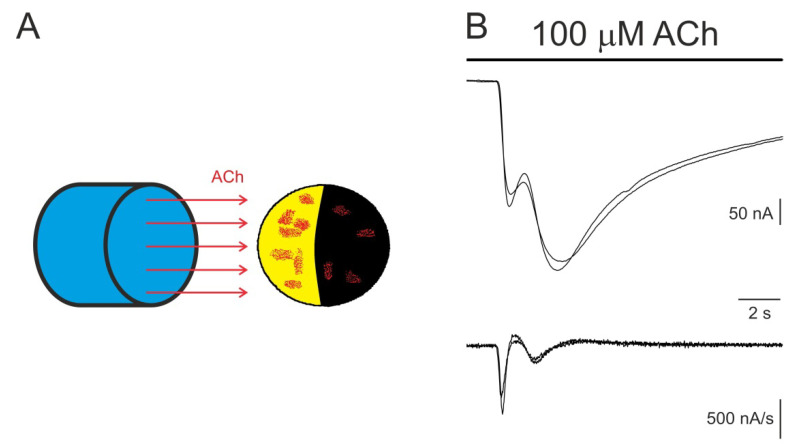
Patchy distribution of microtransplanted nAChRs on the oocyte surface. (**A**) Scheme showing patches of microtransplanted nAChRs on the oocyte surface (red spots) to be activated by the ACh solution superfused from a nearby tube. (**B**) Two superimposed *I_ACh_*s showing at least two sizeable “humps” (more evident in the derivatives of *I_ACh_* records shown below), which were most likely due to the subsequent activation of large patches of nAChRs.

Proteoliposomes bearing reconstituted membrane proteins are microinjected focally into the oocyte, usually at the vegetal hemisphere, to avoid damaging the large nucleus of these cells, located close to the animal pole. Thus, the question is whether the inserted proteins incorporate to the oocyte membrane close to the injection site or, instead, they spread along the oocyte surface. To answer this question, two different experimental approaches have been used: (i) focal recordings mapping the oocyte surface, looking for activity of the transplanted receptors [67]; and (ii) imaging the microtransplanted receptors on the oocyte surface by using fluorescent probes [12,34,47,62,69]. Both techniques showed that either the plasma membranes or purified and reconstituted receptors incorporated in patches into the oocyte membrane, with the density and size of the patches being larger close to the injection site, but they were widely distributed along the oocyte surface. It has been estimated that the total extent of the patches of fluorescent-stained plasma membrane might reach up to 20% of the oocyte surface [62]. In concordance with these observations, functional patches of nAChRs could be found in the animal hemisphere after injecting proteoliposomes at the vegetal hemisphere [67]. This patchy incorporation of the microtransplanted nAChRs could also be evidenced by superfusing the whole oocyte with ACh while recording the *I_ACh_*s. As shown in Figure 4, the elicited *I_ACh_*s commonly showed one or several “humps” during their activation phase, most likely due to the successive activation of patches located around the oocyte surface.

#### 3.1.3. Proteoliposomes of Different Lipid Composition Allow Functional nAChR Microtransplantation

The microtransplant of purified and reconstituted membrane proteins to oocytes allows these proteins to be embedded in specific lipid matrices. The relevant role played by the lipid composition in the mechanisms of membrane fusion is well-established. Furthermore, lipids are crucial in both determining the membrane structure and dynamics and in modulating membrane proteins by specific interactions [46,77,78,79]. Thus, purified nAChRs were reconstituted in different lipid matrices including mixtures of asolectin (Aso), neutral phospholipids such as phosphatidylcholine (PC), anionic phospholipids such as phosphatidic acid (PA), and cholesterol (Chol), and the resulting proteoliposomes were injected into the oocytes. These experiments showed that nAChRs embedded in diverse lipid matrices were functionally transplanted to the oocyte membrane ([68]; see below). This is a quite interesting finding, since nAChRs reconstituted in vesicles of pure PC lost their ability to support cation channel activity [80,81] (reviewed in [82,83]). Therefore, it seems that nAChRs microtransplanted to the oocyte become surrounded, at least in part, by lipids from their own cell membrane. Consequently, when considering the efficiency of functional nAChR transplantation, the role played by the reconstitution lipid composition in the proteoliposome-oocyte membrane fusion is somehow overshadowed by the subsequent lipid exchange taking place around the microtransplanted protein, which is capable, in some way, of customizing its environment, likely by the lateral segregation of specific lipids [78,82,84,85].

### 3.2. Advantages and Limitations of Transplanting Purified Proteins Reconstituted in Artificial Lipid Bilayers

An additional advantage of using *Xenopus* oocytes as the host cell for the functional and biophysical studies of heterologous proteins is that their membrane lipid composition is well-known [86,87], and furthermore, it can be customized to some extent. Thus, the Chol to phospholipid (PL) molar ratio (Chol/PL) in the oocyte membrane (roughly 0.5) can almost be duplicated by incubating oocytes in a solution containing Chol-enriched liposomes. Interestingly, Chol plays a key role in the function of nAChRs, and even Chol rich microdomains may promote cooperativity between neighboring receptors [78,79,88,89]. In fact, *Torpedo* postsynaptic membranes are particularly rich in Chol [78,79,90] and Chol is required for normal functionality of this receptor [91,92,93]. Conversely, the Chol/PL ratio can be decreased, for instance, by incubating oocytes with methyl-β-cyclodextrin [94]. Likewise, the membrane lipid content in the oocyte membrane can be altered by either incubating oocytes with lipid-defined liposomes or activating specific pathways of lipid metabolism. When changing the membrane lipid composition, it should be considered that certain lipids are charged molecules and hence their presence in the oocyte membrane might modulate the function of some microtransplanted proteins, particularly ion channels, by specific interactions (see below), but also by electrostatic mechanisms. Therefore, it is well-known that the biophysical properties of ion channels can be modulated by electrostatic charges present either in the protein itself or in nearby molecules (i.e., the surrounding phospholipids) [70,95,96]. 

**Figure 5:**
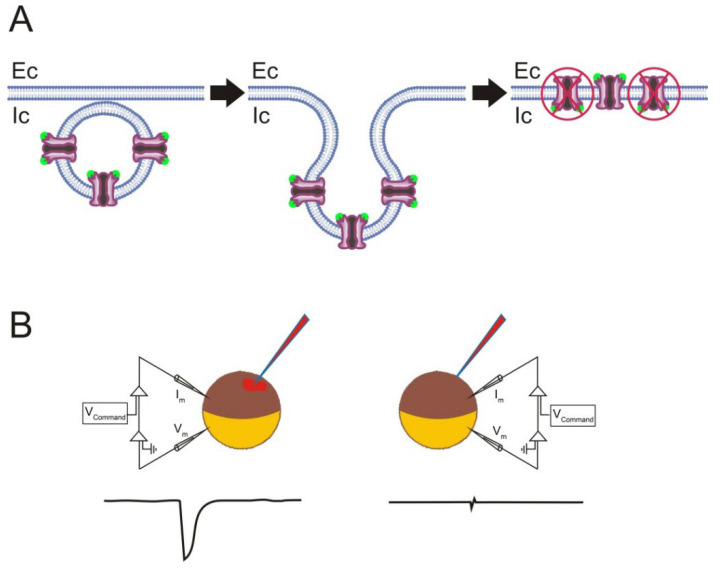
Protein function depends on its adequate orientation in the membrane after microtransplantation. (**A**) Scheme of the putative orientation in the plasma membrane of the microtransplanted nAChRs after intracellular injection of proteoliposomes bearing this protein. Note that the orientation of nAChRs in the injected proteoliposomes was mostly outside-out (left; ACh-binding sites shown as green spheres), whereas it turned to the outside-in orientation after the proteoliposome fused with the plasma membrane (right-side panel). nAChRs adopting a “wrong” orientation have been crossed out; Ec and Ic indicate extracellular and intracellular sides of the membrane, respectively. (**B**) nAChRs with an outside-in orientation lack functional activity, as evidenced by the lack of response when ACh was injected intracellularly (right panel). In contrast, focal ACh pulses over the oocyte surface, applied to nAChRs with the outside-out orientation, elicited *I_ACh_*s (left).

There are also certain handicaps related to this technique of microtransplanting purified and reconstituted proteins to the *Xenopus* oocyte membrane including:

(a) Incorrect orientation of the incorporated proteins in the oocyte plasma membrane. Although the microtransplantation of foreign membrane proteins allows several million nAChRs to be functionally incorporated, the overall efficiency of this approach is, at least for certain proteins, rather low, considering the amount of injected protein [33,67]. Noticeably, membrane proteins of different sizes and shapes might generate proteoliposomes with different protein orientations during the process of detergent dialysis [97]. Therefore, it could be that some of the microtransplanted nAChRs were incorporated into the oocyte membrane in the “wrong” orientation, hence being functionally silent (see Figure 5). It is possible to determine the orientation of the microtransplanted nAChRs in oocytes by combining electrophysiological and binding techniques. These experiments showed the following. (i) Roughly 90% of the microtransplanted nAChRs were incorporated into the oocyte membrane with the binding domain facing up to the cytoplasm [33]. This outside-in orientation of nAChRs in the oocyte membrane could account for the low efficiency of functional nAChR microtransplantation, since nAChRs incorporated with the “wrong” orientation lost their functional activity (Figure 5; [67]). Interestingly, most nAChRs in proteoliposomes are oriented outside-out [91], but fusion of proteoliposomes with the cell membrane commonly results in an inverted protein orientation (see Figure 5; [98]). (ii) The proportion of the incorporated versus injected nAChRs, determined at a fixed time, reached up to 3%, indicating that the fusion of proteoliposomes with the cell membrane is quite an efficient process [33]. Moreover, other membrane proteins such as *Torpedo* Cl^−^ channels (ClC0) were functionally incorporated in the oocyte plasma membrane much more efficiently than nAChRs [33]. Remarkably, a protein change in orientation within the lipid bilayer (protein-flipping) can be triggered, at least in certain proteins, by modifying the lipid composition of the bilayer, both in vitro and in vivo [99].
(b) Presence of multilamellar proteoliposomes. Proteoliposomes bearing purified membrane proteins are commonly obtained by detergent removal dialysis [67,69]. Many of the proteoliposomes harvested under these experimental conditions are multilamellar, particularly when large proteins such as the nAChR are incorporated into liposomes. This constitutes a handicap since the presence of multilamellar proteoliposomes might affect both the protein function and its incorporation to the oocyte plasma membrane. To diminish this handicap, the harvested multilamellar proteoliposomes should be broken into smaller unilamellar vesicles, prior to injecting the sample into the oocytes. This can be attained by sonication of the sample in a water bath until the suspension becomes homogenized and less turbid [67,100,101]. Alternatively, small unilamellar proteoliposomes of homogenous sizes can be obtained from multilamellar vesicles by using a mini-extruder [102].
(c) Microtransplanted proteins do not always remain surrounded by the original proteoliposome lipids. As above-mentioned, some lipid exchange might take place around the protein, as evidenced by the functional recovery of nAChR reconstituted in PC when microtransplanted to the oocyte membrane.

## 4. Microtransplanting nAChRs to the *Xenopus* Oocyte Membrane as a Useful Approach to Study Lipid–Protein Interactions

**Figure 6:**
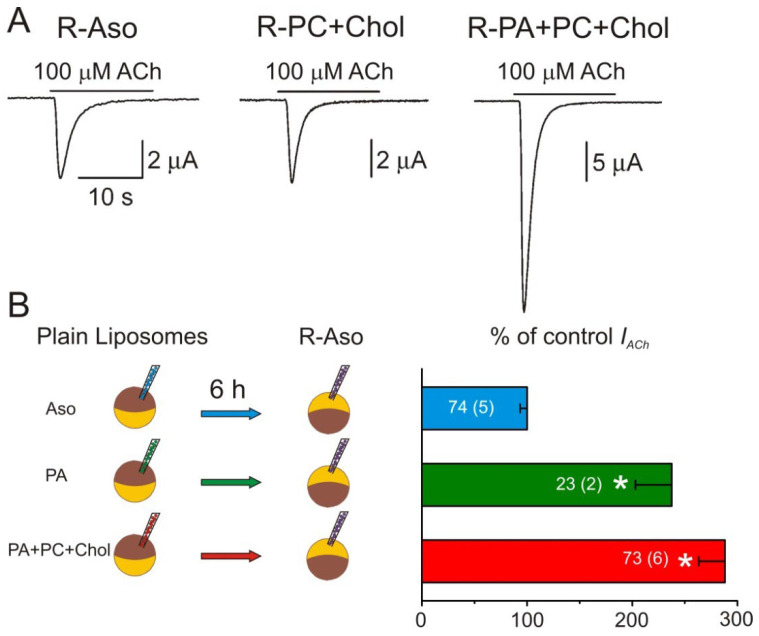
Modulation of nAChR function by specific lipids. (**A**) *I_ACh_*s elicited in oocytes injected with proteoliposomes bearing nAChRs reconstituted in either asolectin (R-Aso), phosphatidylcholine (PC) and Chol (R-PC+Chol), or phosphatidic acid (PA), PC, and Chol (R-PA+PC+Chol). Notice the larger *I_ACh_* in the R-PA+PC+Chol oocyte. (**B**) Effect of pre-injecting the oocyte with liposomes of different composition (Aso, PA, or PA+PC+Chol) 6 h before microinjecting proteoliposomes bearing nAChRs reconstituted in Aso (R-Aso). Left, the scheme shows the experimental procedure. Right, column bars comparing the relative amplitude of the *I_ACh_*s of the indicated groups with respect to the control values (in oocytes pre-injected with Aso liposomes).

The relevance of the interaction between proteins and their surrounding membrane lipids has been reinforced in the last few decades. As above-mentioned (see Section 3.1.3), several in vitro studies have shown that nAChRs are fully functional when reconstituted in a heterogeneous mixture of phospholipids such as that provided by crude soybean (Aso) lipids. Furthermore, these studies indicate that PA and Chol play a major role in preserving the functional activity of this protein. Conversely, when nAChRs are reconstituted in plain PC bilayers, their functional activity is completely lost [80,81,91] (reviewed in [83]), even with Chol restoration [81,92,93]. Taking advantage of the fact that nAChRs can be microtransplanted to the *Xenopus* oocyte membrane after intracellular injection of proteoliposomes, it is possible to determine the effect of the reconstitution lipid matrix on the functional properties of the transplanted nAChRs. Thus, nAChRs were reconstituted in either asolectin (R-Aso), PC:Chol (75:25 molar ratio; R-PC+Chol) or a mixture of PA:PC:Chol (25:50:25 molar ratio; R-PA+PC+Chol). Oocyte injection of nAChR proteoliposomes reconstituted in any of these lipid matrices rendered functional nAChRs in the oocyte membrane (Figure 6A). Interestingly, *I_ACh_*s were significantly larger in oocytes injected with R-PA+PC+Chol than when injected either with R-Aso or R-PC+Chol (Figure 6A; [68]). These larger *I_ACh_*s in R-PA+PC+Chol injected oocytes might be due to either an enhanced fusion of these proteoliposomes with the oocyte plasma membrane or to an increased activity of the nAChRs when surrounded by PA+PC+Chol lipids. To assess whether this *I_ACh_* increase is due to a direct effect of the lipid matrix, some oocytes were pre-injected with plain liposomes of either Aso (control liposomes), egg-PA (PA), or a mixture of PA:PC:Chol (25:50:25) 6 h before injecting into the oocyte R-Aso proteoliposomes (see Figure 6B). Noticeably, the oocytes pre-injected with either PA or PA:PC:Chol elicited larger *I_ACh_*s than those of control oocytes (injected with Aso-liposomes), indicating that PA modulates nAChR function [68,92]. In agreement with this, it is remarkable that the nAChR induces, by itself, the formation of specific PA-rich lipid domains in the membrane vesicles [85]. Moreover, this hypothesis is further supported by the fact that the more nAChR is purified from *Torpedo* electroplaques, the greater the PA proportion is obtained in the membrane fraction, increasing from 0.5 to 1.6% up to 2.2–2.9% of the total lipids [90]. Interestingly, PA is known to enhance the function of other ion channels, as the KcsA, a potassium channel from the *Streptomyces lividans* [83,103,104]. In addition, an enhanced fusion of R-PA+PC+Chol proteoliposomes with the cell membrane was discarded because the incorporation of ClC-0 channels (a minor contaminant in some of the injected samples; [33]) was similar when the oocytes were injected with either the R-Aso, R-PC+Chol, or R-PA+PC+Chol proteoliposomes [68]. 

As above indicated, nAChRs reconstituted in the liposomes of just PC:Chol lacked functional activity [81], but these receptors recovered their full-activity as ion channels after injecting these proteoliposomes into the oocyte (Figure 6A). This functional recovery suggests that the system is sufficiently dynamic to allow for the exchange of lipids between the proteoliposome and their own oocyte membrane. Noticeably, the oocyte plasma membrane has a higher proportion of Chol than phospholipids [45,46], and Chol plays an essential role in preserving nAChR function [89]. Then, why did the oocytes injected with nAChR reconstituted in PA display larger currents? This is most likely because of the tight binding of PA to nAChR, hindering its free exchange with other bulk membrane lipids [90], even leading to the formation of a PA microdomain around the nAChR [85]. This suggested explanation implies that PA acts as a positive modulator of nAChR, enhancing its functional activity [68]. 

## 5. Use of Microtransplanted Proteins to Study Functional and Pharmacological Properties of Ion Channels and Receptors

Transplanting fully-processed foreign membrane proteins to the *Xenopus* oocyte membrane constitutes a valuable approach to carry out detailed functional and biophysical studies on neuroreceptors and other ion channels, preserving their native conformation. After confirming that purified and reconstituted nAChRs microtransplanted to the oocyte membrane preserve their native biophysical properties [67], this technique has been used to study the modulation of this complex protein by different molecules of therapeutic relevance. Thus, this experimental approach has been used to assess the allosteric modulation of muscle-type nAChR function by several cholinesterase inhibitors [105,106], local anesthetics such as lidocaine [107] or akin molecules [59,60], tetracaine [108], and benzocaine [109] or natural anti-inflammatory agents such as peimine [110]. By combining electrophysiological and virtual docking assays, it has been possible to identify specific nAChR residues interacting with different drugs, which support the open-channel blockade elicited by either lidocaine or diethylamine, a molecule resembling the hydrophilic moiety of lidocaine [59,107,110] or tetracaine [108]. Although the kinetic changes elicited by superfusing *Xenopus* oocytes with modulating drugs are rather slow (usually over 1 s; see above the section of drawbacks of using *Xenopus* oocytes to study membrane proteins), the kinetics of the open-channel blockade can be resolved at much higher resolution. Since quaternary-ammonium cholinesterase inhibitors and many local anesthetics are positively charged molecules, the application of positive voltage pulses removes the drug from the channel, thus unplugging the pore by electrostatic repulsion. If the oocyte is maintained in the presence of ACh and the open-channel blocker, the sudden return of the cell membrane potential to negative values, after the application of a strong positive pulse to vanish the open-channel blockade, allows one to determine the kinetics of open-channel blockade with a fairly high temporal resolution [108,110]. Furthermore, the fraction of the voltage field (δ), sensed by the drug at its binding site, can be computed by using the Woodhull equation [111] to electrophysiologically estimate the loci where these open-channel blockers bind within the channel pore [106,107]. Several of the therapeutic molecules tested boosted nAChR desensitization, for instance, tetracaine, most likely acting on residues that are shallowly located in the channel pore [108]. Noteworthy, changes in nAChR desensitization are easier to compute by using oocyte voltage-clamping than by single-channel recording. Since desensitization increases the affinity of nAChR to ACh, the extent of desensitization can be easily determined by comparing the deactivation kinetics of *I_ACh_*s when the modulatory drug is either present or absent [108,110]. In addition, some of these drugs act on nAChRs outside the channel pore, mostly at either the extracellular domain or the transmembrane domain at the inter- or intra-subunit crevices. Drug binding to this loci located outside the channel pore can account for the closed-channel blockade, usually elicited at concentrations higher than those required for open-channel blockade [107,108,110]. 

Remarkably, plasma membranes from either healthy or diseased human postmortem brains (from tissue banks) and other tissues have been successfully microtransplanted to the *Xenopus* oocyte membrane, where the functional and biophysical properties of their neurotransmitter receptors and other ion channels can be studied in detail [12,52,58,66]. Furthermore, this new approach makes the microtransplantation of fully-processed membrane proteins a valuable method to gain insights into the knowledge of the molecular alterations present in specific neurological disorders including Alzheimer, epilepsy, autism, or amyotrophic lateral sclerosis [13,49,53,54,55,56,57,58,65], but also as a powerful and specific way to explore the effects of putative therapeutic molecules and newly designed drugs on native human targets [12,58]. In addition, the microtransplantation procedure has also been probed as a toxicologically-relevant ex vivo assay. Therefore, the effects of neurotoxic insecticides such as DDT (dichlorodiphenyltrichloroethane) on the voltage-dependent Na^+^ channels from rat brain membranes have been successfully tested [34]. A striking advantage of this methodology is that not only can the properties and behavior of the matured membrane proteins be easily studied with diverse techniques, they can also be functionally characterized together with the lipids and other proteins that constituted its natural environment, which might alter or modulate the transplanted protein properties [44].

Although the study of foreign membrane proteins microtransplanted to the *Xenopus* oocyte suffer from certain handicaps, as stated above, this procedure constitutes, as of today, one of the finest approaches to carry out detailed functional studies on native, fully-processed, heterologous membrane proteins from different sources including human tissues and, furthermore, it constitutes an exceptional test bank to assay new medicinal drugs. 

## 6. Conclusions and Future Perspectives

*Xenopus* oocytes have become a valuable tool to study the function and modulation of foreign proteins for over four decades and, most likely, they will continue to be worthy for this purpose for many more years. Thus, there are still many relevant biological problems unsolved that can be best addressed by microtransplanting foreign membrane proteins to this host cell, where they can be more easily studied. For instance, (i) to unravel the modulating effects of specific membrane-lipids on the function of different membrane proteins including pumps, receptors, and ion channels. Actually, thus far, most of the studies dealing with the functional and structural modulation of neuroreceptors and ion channels by their surrounding lipids have been carried out on artificial membranes. Thus, the introduction of novel methods is required to study the lipid–protein interactions in native cell membranes, which might confirm, or not, the previous results found in artificial systems. (ii) To explore the targeting of foreign proteins to the cell membrane, which might be particularly relevant for mutated proteins and the scaffolding by other molecules surrounding the mature membrane protein. (iii) To assess the mechanisms of the dysfunction of membrane proteins obtained from pathological tissue banks. Actually, transplanting receptors and ion channels from human post-mortem tissues from individuals either healthy or suffering specific diseases might be considered as a “Rosetta stone” for the analysis of human membrane protein function [112], and the transplanted proteins should constitute an exceptional target for assaying new molecules designed to modulate specific proteins. (iv) To gain a deeper insight into the mechanisms by which new targeted molecules modulate the function of transplanted membrane proteins. (v) As a cell model to study the mechanisms underlying the constitutive fusion of membranes. (vi) As a suitable cell to study mutated receptors or ion channels overexpressed in convenient cell lines.

Although our knowledge on the structure, function, and modulation of neuroreceptors and ion channels in healthy and pathological tissues has been greatly expanded over the last decades, many unanswered questions remain and, most likely, the *Xenopus* oocytes will continue helping to provide valuable responses. 

## Data Availability

Not applicable.

## Abbreviations

Ach	Acetylcholine;
AMPA	α-amino-3-hydroxy-5-methyl-4-isoxazolepropionic acid;
BAPTA-AM	1,2-bis (2-aminophenoxy)ethane N,N,N′,N′-tetraacetic acid acetoxymethyl ester
ClC0	Chloride channel type 0 (*Torpedo*)
Chol	Cholesterol
Chol/PL	Cholesterol to phospholipid molar ratio
δ	Fraction of the voltage field sensed by a drug in the membrane
DDT	Dichlorodiphenyltrichloroethane
EGTA	Ethyleneglycol-bis (β-aminoethylether)-N,N,N′,N′-tetraacetic acid
Ec	Extracellular side
EC_50_	Half maximal effective concentration
GABA_A_R	Gamma-aminobutyric acid receptor type A
HEK (HEK 293)	Human embryonic kidney 293 cells
*I_ACh_*	ACh-elicited current
IC_50_	Half maximal inhibitory concentration
InsP3	Inositol 1,4,5-trisphosphate
Ic	Intracellular side
[Ca^2+^]_i_	Intracellular Ca^2+^ concentration
KCC1	K^+^–Cl^−^ cotransporter type 1
nAChR	Nicotinic acetylcholine receptor
NMDA	N-methyl-D-aspartate
PA	Phosphatidic acid
PC	Phosphatidylcholine
KcsA	A potassium channel from the Streptomyces lividans
R-Aso	Proteoliposome of asolectin bearing nAChRs
R-PA+PC+Chol	Proteoliposome of PA, PC and Chol bearing nAChRs
R-PC+Chol	Proteoliposome of PC and Chol bearing nAChRs
NKCC2	Na^+^–K^+^–Cl^−^ cotransporter type 2
TMEM16A	Ca^2+^ activated Cl^−^ channel, anoctamin1
